# Access to Hepatitis C Treatment during and after Incarceration in New Jersey, United States: A Qualitative Study

**DOI:** 10.3390/life13041033

**Published:** 2023-04-17

**Authors:** Samir Kamat, Sankeerth Kondapalli, Shumayl Syed, Gabrielle Price, George Danias, Ksenia Gorbenko, Joel Cantor, Pamela Valera, Aakash K. Shah, Matthew J. Akiyama

**Affiliations:** 1 Department of Medical Education, Icahn School of Medicine at Mount Sinai, New York, NY 10029, USA; 2 Rutgers New Jersey Medical School, Newark, NJ 07103, USA; 3 Institute for Healthcare Delivery Science, Mount Sinai Health System, New York, NY 10016, USA; 4 Center for State Health Policy, Rutgers University, New Brunswick, NJ 08901, USA; 5 Department of Urban-Global Public Health, Rutgers University School of Public Health, Newark, NJ 07102, USA; 6 Community Health Justice Lab, Newark, NJ 07107, USA; 7 Department of Emergency Medicine, Department of Psychiatry and Behavioral Health, Hackensack Meridian School of Medicine, Nutley, NJ 07110, USA; 8 Department of Medicine, Divisions of General Internal Medicine and Infectious Disease, Montefiore Medical Center, Albert Einstein College of Medicine, Bronx, NY 10461, USA

**Keywords:** HCV, Hepatitis C, treatment, direct-acting antiviral DAA, prison, jail, incarceration, linkage to care, qualitative

## Abstract

Despite effective antiviral therapy for hepatitis C virus (HCV), people who are incarcerated and those returning to the community face challenges in obtaining HCV treatment. We aimed to explore facilitators and barriers to HCV treatment during and after incarceration. From July–November 2020 and June–July 2021, we conducted 27 semi-structured interviews with residents who were formerly incarcerated in jail or prison. The interviews were audio-recorded and professionally transcribed. We used descriptive statistics to characterize the study sample and analyzed qualitative data thematically using an iterative process. Participants included five women and 22 men who self-identified as White (*n* = 14), Latinx (*n* = 8), and Black (*n* = 5). During incarceration, a key facilitator was having sufficient time to complete HCV treatment, and the corresponding barrier was delaying treatment initiation. After incarceration, a key facilitator was connecting with reentry programs (e.g., halfway house or rehabilitation program) that coordinated the treatment logistics and provided support with culturally sensitive staff. Barriers included a lack of insurance coverage and higher-ranking priorities (e.g., managing more immediate reentry challenges such as other comorbidities, employment, housing, and legal issues), low perceived risk of harm related to HCV, and active substance use. Incarceration and reentry pose distinct facilitators and challenges to accessing HCV treatment. These findings signal the need for interventions to improve engagement in HCV care both during and after incarceration to assist in closing the gap of untreated people living with HCV.

## 1. Introduction

Hepatitis C virus (HCV) is a bloodborne infection that, if untreated, may lead to liver damage, hepatocellular carcinoma, and cirrhosis. HCV prevalence is 10- to 20-fold greater in incarcerated populations than in the general population, with 17.4% of incarcerated individuals having positive HCV serology [1]. Treatment has vastly improved following the emergence of direct-acting antivirals (DAAs) over the last decade. DAAs have a short duration of about 8–12 weeks, high rates of viral clearance of around 90–100%, and minimal side effects. Despite formative advancements in treating HCV and the convenience of these treatments, the wider uptake among people who are incarcerated remains limited [2]. 

The U.S. carceral system is divided into prisons and jails. In prisons, sentences are typically greater than one year, whereas jails are shorter-term facilities with lengths of stay that can be unpredictable, ranging from just several hours to months depending on whether a detainee is awaiting trial or has received a short sentence. The division of the carceral system into prisons and jails has implications for HCV treatment because longer sentences may help to facilitate HCV treatment by reducing the risk of treatment interruption in the transition to the community. Despite challenges associated with shorter-term jail stays, the feasibility of HCV treatment has been demonstrated [3,4,5].

The HCV care cascade typically includes screening, confirmation of HCV viremia, clinician connection, treatment initiation, and confirmation of viral clearance at 12 weeks post-treatment [3]. Screening comes as a standard or rapid immunoassay for antibodies, HCV RNA, and genotype assay; within carceral settings, this can manifest through universal opt-out screening or is based on risk factors [3]. Treatment typically occurs at local hospitals for assessment and treatment, in-reach services, or telemedicine consultations [3].

Several factors have been documented that limit HCV treatment uptake in the criminal legal system. Structural-level barriers include limited carceral healthcare budgets, political will, and the workforce capacity to provide DAA therapy [6,7]. At the patient level, poor HCV-related knowledge, stigma, medical mistrust, low social support, and concern for relapse to active drug use and HCV reinfection have been identified as barriers to the DAA treatment uptake [8,9,10]. Having a routine, better motivation, and peer support have been documented as facilitators [9,11]. After incarceration, individuals face different barriers in linking to HCV care such as inadequate insurance coverage, relapse to active substance use, and competing priorities, such as other medical comorbidities and unstable housing [12]. This is evidenced by a study demonstrating that few individuals initiated treatment and were cured even at a transition clinic, which specializes in providing medical care to formerly incarcerated individuals, including treating chronic HCV infection [13]. 

To date, few studies have elicited patient perspectives on what impedes and facilitates HCV treatment both during and after incarceration. The U.S. has the highest incarceration rate of any country in the world, with a high rate of syndemic substance use disorder and HCV. Patient perspectives are crucial to understanding the experience of individuals in carceral settings and the continuity of HCV care following reentry to the community. To address this gap, we conducted semi-structured interviews with formerly incarcerated people living with HCV in New Jersey (NJ), United States, to identify barriers and facilitators to HCV treatment uptake during and after incarceration.

## 2. Materials and Methods

### 2.1. Setting

In NJ, USA, nearly 40,000 individuals are incarcerated at any given time, including almost 20,000 in state prisons, 15,000 in county jails, and 3200 in federal prisons [14]. In 2011, 10,835 prisoners were released from the state’s carceral facilities, and within three years following release, 53% were rearrested, 40% were reconvicted, and 31% were re-incarcerated [15]. Given the high rate of HCV among incarcerated populations, these data suggest a sizeable population having HCV, including those presently incarcerated and those returning to the community.

To draw insights on the facilitators and barriers to treatment among these populations, we conducted in-person, one-on-one, semi-structured interviews at a statewide reentry program between July–December 2020 and June–July 2021.

### 2.2. Recruitment

Reentry program staff identified and referred eligible clients using convenience sampling for in-person screening and interviews. The reentry program serves individuals who are 18 and over who have been court-involved at any point in their lives on all-around needs related to successfully reintegrating into their communities. We included individuals who were: (1) greater than 18 years old; (2) residing in NJ, US; (3) incarcerated within the past five years; (4) self-reported to have an HCV diagnosis; and (5) willing to provide informed consent.

### 2.3. Interviews

We developed a semi-structured interview guide based on prior qualitative studies among people living with HCV to understand participants’ experiences with HCV testing, diagnosis, and treatment during and after incarceration [16,17,18,19]. Examples of interview questions included: Tell me about the way in which you were offered HCV treatment. What made it challenging to access HCV treatment? How has having HCV affected your life? What do you wish people understood about having HCV in prison or jail? We also incorporated a sociodemographic survey at the end of the qualitative interview.

At the scheduled session, we screened each client for inclusion, obtained informed consent, and a male medical student (SK) with training in patient history taking and qualitative research conducted face-to-face interviews in private spaces within the reentry program site, lasting anywhere from 30–60 min. We provided participants with a $25 gift card for their participation. We conducted interviews until thematic saturation was achieved [20].

### 2.4. Analysis

Once all the interviews were conducted and transcribed, we analyzed qualitative data in an iterative process using a thematic analysis [21] to identify the facilitators and barriers to HCV care during and after incarceration. Three investigators (SK, SS, SK) developed a coding scheme to become familiar with the data by open-coding five transcripts. After each transcript, the coding scheme was further refined to reflect the content and emergence of new themes. After completing the five transcripts, we developed a codebook in consultation with content experts (KG, MJA, AS) that we applied to all transcripts. One investigator coded each transcript, including re-coding the initial five, and another reviewed the applied codes and noted any discrepancies. We held weekly meetings to review and resolve any discrepancies and reached consensus on the applied codes. A third author (KG) adjudicated during cases of disagreement or lack of consensus. We completed an additional analysis by reviewing existing codes and excerpts from transcripts to develop analytic memos [22], which summarized information in the codes and were used to contextualize emerging themes. We include the participant’s age and sex for all illustrative quotes. We reported findings in line with the Consolidated Criteria for Reporting Qualitative Research (COREQ) guidelines and used NVIVO 12 for analysis. 

We used descriptive statistics to characterize the study sample. Frequencies and proportions were calculated for categorical data, and we calculated means, medians, and ranges for continuous data. The Rutgers University Institutional Review Board approved this study.

## 3. Results

Participants were predominantly male (*n* = 22) who self-identified as White (*n* = 14), Latinx (*n* = 8), or Black (*n* = 5) and were incarcerated a median of 5 and a mean of 11.5 times. Their most recent incarceration lasted anywhere from 5 days to 30 years, with a mean of 3.7 years and a median of 5 months. A total of 10 participants had received prior HCV treatment (Table 1).

**Figure 1:**
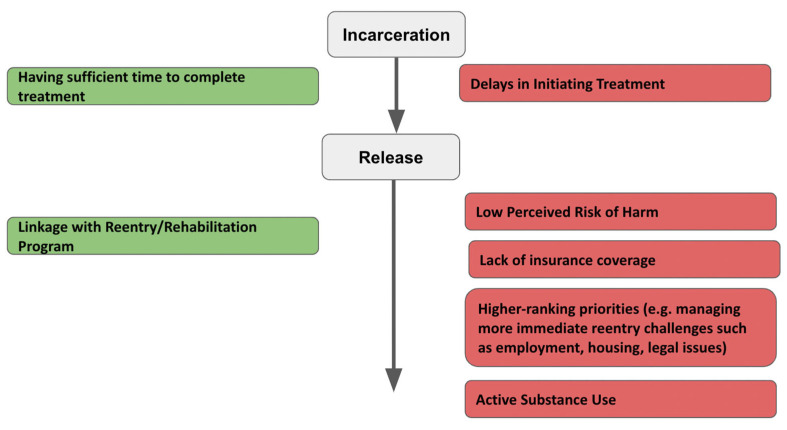
Summary of themes related to facilitators and barriers to accessing HCV care during and following incarceration.

We identified distinct themes on the facilitators and barriers to HCV treatment during and after incarceration (Figure 1). During incarceration, a key facilitator included having sufficient time to complete treatment and the corresponding barrier was delaying the treatment initiation. Facilitators following incarceration included linkage with reentry programs. Barriers included a lack of insurance coverage, higher-ranking priorities, a low perceived risk of harm, and active substance use.

### 3.1. Facilitators & Barriers to Accessing Treatment during Incarceration

#### 3.1.1. Having Sufficient Time While Incarcerated for HCV Treatment 

While HCV treatment with DAA therapy can now be completed in 8–12 weeks, many individuals incarcerated in jails are not detained for this long. Moreover, evaluation by an HCV-treating provider and pre-treatment workup can extend the duration of time required for HCV treatment initiation. Such factors often lead to difficulty initiating and completing treatment, even where DAAs are available.

*“I wasn’t there long enough to get the treatment. So they wasn’t offering [it to] me. I was only there for 90 days.”*

(65-year-old male)

Some reported being actively discouraged by the inability to initiate HCV treatment while incarcerated, and that it was a missed opportunity given that periods of incarceration are a time with fewer competing priorities than during the transition to the community.

*“I think the better time would have been when I was actually in prison…because I don’t have that many responsibilities.”*

(33-year-old male)

Another participant who was treated while incarcerated detailed DAA treatment regimens and the associated adherence necessary for treatment success. He described why the prison environment was optimal for his adherence.

*“It’s a 12-week system… You had to take the pill every day. You can’t miss no days.”*

(57-year-old male)

Some participants offered a solution to the issue of withholding treatment for incarcerated individuals if their stays were too short, with one suggesting a promising role for carceral settings to promote treatment by linking patients with community providers.

*“They can coordinate with an outside [organization]…say…somebody started treatment behind the wall, but then they’re released that they have certain things in place that they can send those people to, to complete that treatment so that they’re not left in limbo of being halfway done in the treatment. And they’re not also discouraged from starting a treatment until they get home.”*

(37-year-old male)

#### 3.1.2. Delays to Initiating Treatment

Many participants reported that there were delays, often beyond their control, for initiating treatment. Most commonly, this was due to restricting treatment based on the severity of liver fibrosis and, therefore, there were long waitlists.

*“My initial reaction was I wanted to get treatment, but they were saying my levels…my enzymes or something aren’t high enough to qualify to get the treatment.”*

(33-year-old male)

Participants with mild disease were less likely to receive treatment than those with severe disease, with some being on waitlists for five years before receiving treatment.

*“No, I waited five years to get the medication approved for me to get it. I was on the waiting list. The way it runs inside of the jail is whoever’s condition is worse. That’s who get treated first, not who applied first. So, it took like almost five years from my time to come to get the treatment.”*

(54-year-old male)

Additional participants added that further delays were due to advancing through the pre-treatment workup (e.g., blood tests, imaging), extending the time to treatment approval:

*“[It’s] too slow between the appointment[s]…two-three months every next appointment, you know? Too slow.”*

(48-year-old male)

For some, the delays and restrictions decreased optimism about pursuing treatment during incarceration. For example, one participant gave up on being treated for this reason and instead opted to pursue treatment once out of incarceration.

*“I gave up on it because it was…far-fetched… I’m in the bottom of the list, they’re not trying to move me up. And even though I had a lot of time to do, I wasn’t going to meet the standards to get to that position.”*

(56-year-old male)

### 3.2. Facilitators & Barriers to Accessing Treatment following Incarceration

#### 3.2.1. Linkage with Reentry Programs (e.g., Halfway Houses or Rehabilitation Programs)

Participants reported positive views about the reentry programs they were participating in after incarceration, stating that they were helpful in seeking and remaining adherent to HCV treatment. These programs made appointments for patients, administered medication on a schedule, and gathered labs, all of which participants appreciated having coordinated for them.

*“I’m glad I got [the treatment] administered on a program… because I knew I couldn’t miss a day because if I didn’t take my medication, they were calling my counselor. My counselor would come get me and make me go and take my medication…It might’ve been a different story if [I] was on the streets because sometimes… you forget to take your medication, you’re not really on point.”*

(37-year-old male)

Several participants also cited additional benefits of reentry programs, such as helping to attain housing and abstaining from substance use.

*“I had to go to like a sober living house. Like I changed my life. It was like one of the best things for me. I feel like a family like, a bond I’m held accountable.”*

(26-year-old male)

#### 3.2.2. Lack of Insurance Coverage

Multiple participants reported that cost was a significant barrier to treatment, and treatment was completely unattainable without insurance.

*“Financially, I’m not stable. I just got out of prison from doing 11 years. So I’ve got to get on my feet. And…sometimes I think, wow, well horizon [Managed Medicaid] or whatever coverage [includes HCV treatment coverage…] I could get, you know, and put a seal to it.”*

(41-year-old male)

Yet, some participants reported obstacles to obtaining Medicaid coverage, which impeded or prevented them from receiving HCV treatment.

*“So I don’t know why [Medicaid] denied me…I had insurance for like eight months. I get my own private insurance, but before I could even get to a specialist, I ended up having to get it canceled because I had to pay my rent. It was so expensive.”*

(49-year-old female)

For some participants, wait times between the application and approval for treatment were protracted, leading to decreased morale.

*“I was in limbo because they were like, okay, well, you’re approved. I’m waiting for the medication. Thinking it’s, okay ...might take a week. It wound up taking like a month to come. So I lost hope.”*

(37-year-old male)

#### 3.2.3. Higher-Ranking, Competing Priorities

Several participants stated that higher-ranking priorities such as other comorbidities, employment, housing, and legal issues took precedence over HCV treatment. For example, coordinating multiple health appointments during the reentry period made obtaining HCV treatment difficult. Some participants noted that they did not have the ability or skills to coordinate their care.

*“People who are addicts are not like strong when it comes to like organizing their life…it’s like a life skill that a lot of us never developed, like organizing our life, making appointments, making phone calls.”*

(32-year-old female)

Participants commonly reported socioeconomic challenges to obtaining HCV treatment such as childcare responsibilities, work responsibilities, lack of adequate transportation, and the cost of going to the doctor.

*“I would work, you know, Monday through Saturday from seven to seven…. it was real hectic to take off a day and go and get it done more likely.”*

(30-year-old male)

#### 3.2.4. Low Perceived Risk of Harm

Multiple participants reported that the perceived asymptomatic nature of the HCV disease course led to a low motivation to pursue treatment.

*“It’s not visual, I mean, you don’t see the symptoms. It’s almost like it’s not there…Out of sight out of mind.”*

(32-year-old female)

Some participants further reported delaying treatment until their disease severity increased.

*“Right now, I’m not sick…like nothing’s bothering me…when something does happen, then I’ll mention it to a doctor.”*

(35-year-old female)

However, some participants believed that cure from HCV improved their stress levels and quality of life despite being asymptomatic.

*“It’s given me a peace of mind that… I got it taken care of… there wasn’t really any like physical side effects…I’m really grateful that it worked out the way it.”*

(37-year-old male)

#### 3.2.5. Active Substance Use

Substance use was deeply intertwined with the linkage to HCV care in the post-incarceration period. Participants stated that active substance use deterred them from seeking treatment:

*“I wouldn’t have wanted it to get treated cause I was just on the run anyway. Not from any police or anything just it’s when you’re in like heavy addiction, you call it like running. So I was just on the run I was just doing my thing. I just wanted one more. I would not have slowed down to get treatment.”*

(26-year-old male)

According to some, being cured of HCV was not enough to stay abstinent from substance use. This was the case for a 28-year-old male re-infected with HCV shortly after finishing the treatment program.

*“I got treated and it was coming up undetected, but then you know I fell back in addiction and I reinfected myself unfortunately... Couldn’t control myself”*

(28-year-old male)

At the same time, participants cited multiple factors that helped them remain abstinent from active substance use, such as one participant who cited her pregnancy and parenthood.

*“Since I got pregnant with [my son], I might have [used]… just a couple of times… But for…the last year I haven’t used really at all. So that’s really what has stopped me from doing It [seeking treatment] would be getting high.”*

(35-year-old female)

## 4. Discussion

Despite advances in HCV treatment, challenges remain in initiating DAA therapy during and after incarceration. Our study identified important facilitators and barriers to accessing HCV care among individuals involved with the criminal legal system in New Jersey. Our findings indicate that HCV treatment is facilitated by having sufficient time to complete it, such as longer stays in jail or prison sentences, and it is impeded by long waitlists and delays during the pre-treatment workup. When transitioning from a carceral facility to the community, the linkage to reentry programs is important for facilitating access to HCV treatment within the community. Challenges in engaging in HCV treatment after incarceration included higher-ranking competing priorities, lacking insurance coverage, a low perceived risk of harm, and active substance use. 

Since their emergence, DAAs have been delivered in carceral settings using prioritization protocols aimed at providing treatment to patients meeting certain criteria such as advanced liver disease [23]. We identified similar barriers in New Jersey including the duration of incarceration and fibrosis restrictions. A retrospective study of unrestricted access to DAAs for incarcerated people with chronic HCV resulted in a 460% increase in treatment access, a higher SVR and treatment completion rate, and a lower loss to follow up compared with restricted access [24]. 

In addition to the duration of incarceration, many individuals are confronted with numerous other competing priorities that may impact their HCV treatment, such as lack of employment, unstable housing, and active substance use. Previous studies have demonstrated that individuals experiencing these competing priorities experience challenges in engaging in medical care [25,26]. Thus, individuals may not link to care without first meeting other needs (e.g., employment, housing, substance use treatment). Respondents attributed much of their success in accessing treatment to reentry programs that provided the necessary stability and programming to initiate treatment. The importance of reentry programs was viewed as a facilitator in connecting to HCV care shared by patients, carceral staff, and policymakers in a qualitative study conducted in Massachusetts [10]. The linkage to care may be further improved by providing transitional care coordination in connecting people living with HCV to care in the community as evidenced by clinical trials conducted in New York and Australia [12,27].

Given that most people living with HCV are asymptomatic, many participants reported that a low perceived risk led to low motivation to pursue treatment. Swan et al. likewise found in a qualitative study among people who inject drugs along the HCV care cascade that respondents assumed the absence of symptoms equated to optimal health, frequently leading to not engaging in HCV care. Conversely, knowledge of potentially life-threatening sequelae or becoming symptomatic was associated with an increased willingness to engage in treatment [28]. In our study, respondents often had a basic understanding of HCV (e.g., affecting the liver). Expanding educational efforts may be one opportunity to increase awareness of HCV, perhaps through peer education or peer support [29]. For example, the New Mexico Peer Education Project utilized peer educators drawn from the general prisoner population, and between 2009 and 2016, 482 peer educators delivered HCV educational outreach to 5066 prisoners [30]. In addition, digital videos have shown promise in providing underserved populations with an improved understanding of disease [31]; in the Rhode Island Department of Corrections, for example, an 8-min educational module was incorporated into the rapid screening for Hepatitis C [32]. 

On a structural level, many respondents seeking HCV treatment encountered various insurance barriers, from having no coverage to experiencing delays in medication approvals, impinged on timely treatment initiation. Therefore, linking release with continued coverage can help individuals access treatment earlier [33]. The present policy proposals aiming for that goal are promising, like the Medicaid Reentry Act, which aims for the reinstatement of Medicaid benefits 30 days before release, though this remains complicated in terms of the operational logistics (coordinating quality and safety, billing, carceral service-delivery models), and is still insufficient for HCV therapy given the duration of treatment [34].

Our findings suggest that one means of linking those in reentry to HCV treatment includes both addressing the competing priorities for treatment and, where possible, simplifying the process of engagement in healthcare. One-stop-shop models, such as the co-location of HCV services with substance use disorder treatment and mobile health units, can help bring the treatment to where individuals are [35,36]. Regarding active substance use in the community, harm reduction should be emphasized to prevent the continued potential spread of HCV or re-exposure. These efforts should include a connection to needle and syringe exchange programs and opioid agonist therapy [7]. Moreover, prior to and following release, discharge planners and patient navigators can help bridge those in reentry to services in the community, helping to manage other competing priorities [7]. For example, peer navigators can help patients connect with a network of different community resources, and also provide key education on HCV [36].

This study has some limitations. First, given its qualitative nature, social desirability might have influenced the participants’ reporting of their lived experiences. While this was primarily mitigated by emphasizing the anonymous and confidential nature of the interviews, this bias is difficult to eliminate. In addition, we used convenience sampling, which may have introduced some bias to our findings. Further, we did not collect structured data on whether the participants were provided discharge planning or assistance with restarting their insurance coverage. Despite these limitations, this study contributes key insights informing the program and policy changes during and after incarceration for people living with HCV.

## 5. Conclusions

Improving HCV treatment uptake and transitions in care will benefit both reducing the individual long-term health consequences of HCV and reducing the spread of this infection among marginalized populations. By integrating insights directly from formerly incarcerated individuals, our data raise opportunities and areas for reform. For example, these findings can inform the development of surveys or a needs assessment of a wider population of justice-involved individuals to direct programming. Future studies should focus on further stakeholder engagement to shape policy and identify ways to improve care for individuals involved with the criminal legal system living with HCV.

## Figures and Tables

**Table 1:** Participant characteristics.

Characteristic	N = 27
Age	Mean: 42, Stdev: 12.2, Range: 24–65
Gender	
Female	5
Male	22
Race	
White	14
Latinx	8
Black	5
Marital Status	
Single	20
Divorced	6
Married	1
Number of prior incarcerations	Mean: 11.5, Median: 5, Range: 1–100
Length of most recent incarceration	Mean: 3.7 years, Median: 5 months, Range: 5 days–30 years
Previous HCV Treatment	10

## Data Availability

De-identified data can be shared upon reasonable request from the corresponding author. The data are not publicly available to preserve participant privacy.
